# A Fluorescent Glucose Transport Assay for Screening SGLT2 Inhibitors in Endogenous SGLT2-Expressing HK-2 Cells

**DOI:** 10.1007/s13659-018-0188-4

**Published:** 2018-11-01

**Authors:** Yan-Ting Lu, Xiu-Li Ma, Yu-Hui Xu, Jing Hu, Fang Wang, Wan-Ying Qin, Wen-Yong Xiong

**Affiliations:** 1 State Key Laboratory of Phytochemistry and Plant Resources in West China, Kunming Institute of Botany, Chinese Academy of Sciences, Kunming, 650201 China; 2 University of the Chinese Academy of Sciences, Beijing, 100049 China; 3 Yunnan Key Laboratory of Natural Medicinal Chemistry, Kunming, 650201 China

**Keywords:** SGLT2 inhibitor, 2-NBDG, HK-2, Glucose uptake

## Abstract

**Abstract:**

The sodium-dependent glucose transporters 2 (SGLT2) plays important role in renal reabsorption of urinal glucose back to plasma for maintaining glucose homeostasis. The approval of SGLT2 inhibitors for treatment of type 2 diabetes highlights the SGLT2 as a feasible and promising drug target in recent years. Current methods for screening SGLT2 inhibitors are complex, expensive and labor intensive. Particularly, these methods cannot directly measure nonradioactive glucose uptake in endogenous SGLT2-expressing kidney cells. In present work, human kidney cells, HK-2, was incubated with a fluorescent d-glucose derivant 2-[*N*-(7-nitrobenz-2-oxa-1,3-diazol-4-yl) amino]-2-deoxy-d-glucose (2-NBDG) and the fluorescent intensity of 2-NBDG was employed to measure the amount of glucose uptake into the cells. By optimizing the passages of HK-2 cells, 2-NBDG concentration and incubation time, and by measuring glucose uptake treated by Dapagliflozin, a clinical drug of SGLT2 inhibitors, we successfully developed a new assay for measuring glucose uptake through SGLT2. The nonradioactive microplate and microscope-based high-throughput screening assay for measuring glucose can be a new method for screening of SGLT2 inhibitors and implied for other cell assays for glucose measurement extensively.

**Graphical Abstract:**

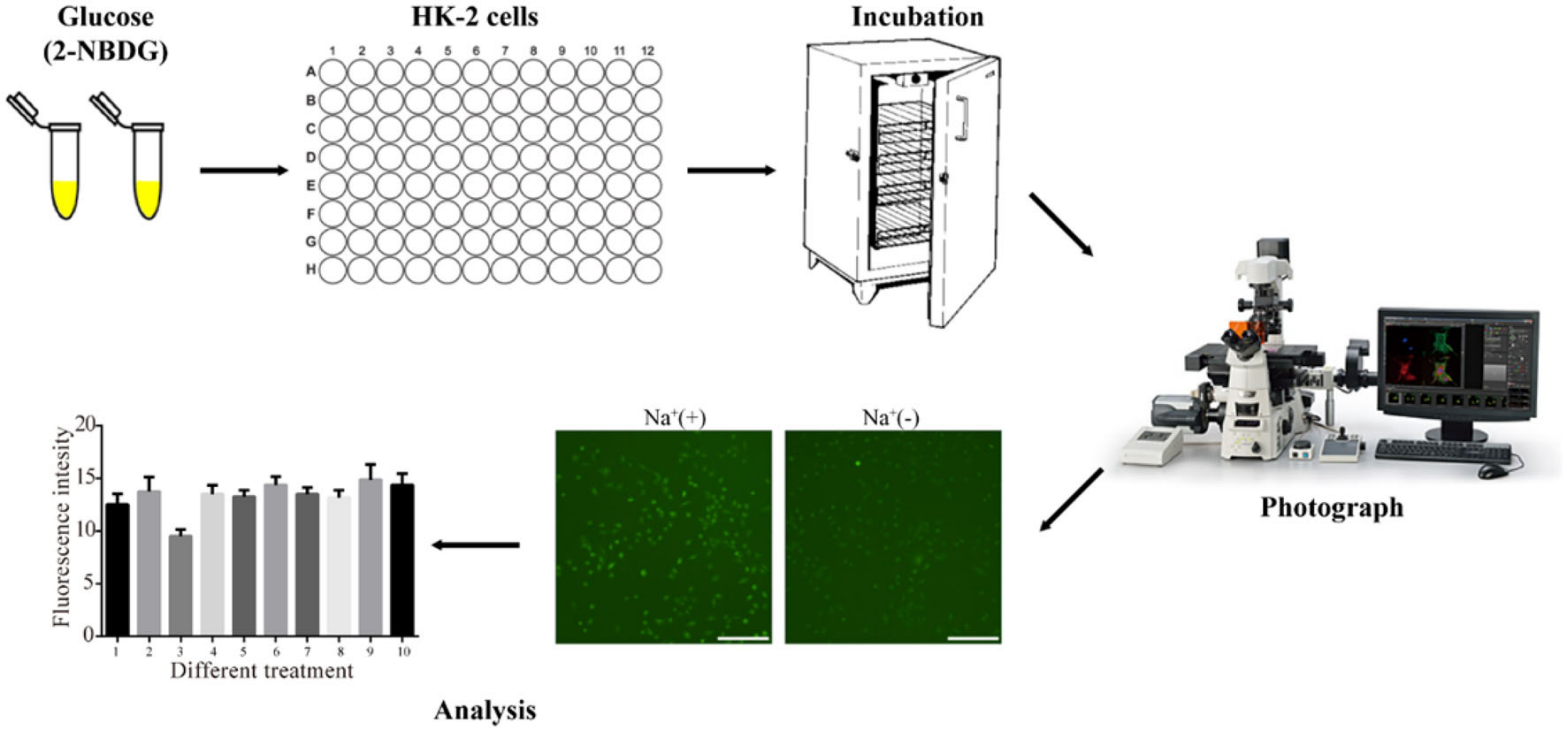

**Electronic supplementary material:**

The online version of this article (10.1007/s13659-018-0188-4) contains supplementary material, which is available to authorized users.

## Introduction

Type 2 diabetes mellitus (T2DM), a metabolic disease characterized by hyperglycemia, is the results of reduced production and/or utilization of insulin, which also plays pivotal roles in the development of several important renal, cardiovascular, and neurological complications [1, 2]. Besides insulin supplement, current therapies for T2DM include oral drugs, such as biguanides, thiazolidinediones, sulfonylureas, α-glucosidase inhibitors, glinides and incretin mimetics [3, 4]. These traditional drugs target the liver, small intestine, adipose tissue, skeletal muscle, and pancreas respectively [4].

Due to their limited efficacy and undesired side effects, the hyperglycemia still cannot be alleviated in many cases. Hence, novel targets and related drugs with new mode of action and well safety are urgently needed. Among these, sodium-dependent glucose transporters 2 (SGLT2) in kidney has been emerged as an attractive target because it is closely involved in T2DM with a distinct mechanism that reduce blood glucose levels independently of insulin secretion. Blood glucose is filtered in the glomerulus and then reabsorbed and returned to the systemic circulation by the proximal tubule of the kidney. Sodium-dependent glucose transporters (SGLTs) are membrane proteins mediating reabsorption of filtered glucose. Hyperglycemia boosts the filtered and reabsorbed glucose up to two- to three-fold, indicating that suppression of glucose reuptake through SGLTs would promote urinary excretion and thus lead to decreased plasma glucose levels [5]. Two major types of glucose co-transporters in SGLTs, SGLT1 and SGLT2, were identified. SGLT1 is primarily localized in the intestine, whereas SGLT2 is mainly expressed in the kidney where it is involved in more than 90% glucose reuptake from the glomerular filtrate [6, 7], and the expression of SGLT2 is correlated with T2DM [8]. Consistent with its function in glucose reabsorption, selective SGLT2 inhibitors indeed increase urinary glucose excretion, leading to reduce plasma glucose levels and lower body weight [1, 9]. To be noted, the SGLT2 inhibitors work independently, albeit without insulin, ameliorate blood glucose control in all stages of T2DM in the absence of clinically relevant hypoglycemia, and could be combined with other antidiabetic drugs. Therefore, The SGLT2 inhibitors have been developed into a new class of antidiabetic agents approved in T2DM [10]. Studies are under way to investigate their use in type 1 diabetes mellitus (T1DM) as well [11, 12]. By lowering diabetic glomerular hyperfiltration and blood pressure, SGLT2 inhibitors may induce protective effects on cardiovascular system and the kidney beyond glycemic control [13, 14].

The first SLGTs inhibitor phlorizin is a natural phenolic O-glucoside that reduces blood glucose through competitively inhibition of human SGLT2 and SGLT1 in real and intestinal [15]. Canagliflozin, Dapagliflozin, and Empagliflozin are currently clinical drugs of SGLT2 inhibitor [16, 17]. They are available as single-ingredient products and also in combination with other diabetes medicines such as Metformin. All these SGLT2 inhibitors similarly induce a sustained urinary glucose loss of 40–80 g/day, associated with good blood glucose-lowering efficacy in T2DM [10, 18]. However, long time use of these drugs may evoke the potential for cancer, liver injury, and/or other unexpected side-effects [19]. So there is still an urgent demand for new diabetes medications targeting SGLT2.

Currently, non-human and non-renal epithelial cells (COS-7 and CHO cell) were mostly used for screening SGLT2 inhibitors. However, human kindney-2 (HK-2) cells is a humanized renal proximal tubular cells which are transfected with human papilloma virus. It retains a phenotype indicative of well-differentiated proximal tubular cells (PTC), such as positive for alkaline phosphatase, cytokeratin, fibronectin, negative for factor VIII-related antigen. Furthermore, HK-2 cells also possess functional characteristics of proximal tubular epithelium including Na^+^-dependent sensitive sugar transport [20]. Given these characteristics, HK-2 cells are proved to be a powerful new tool for the study of PTC and it have been well used to explore the mechanisms of kidney injury and repair [21, 22]. Therefore, HK-2 cells could potentially be implemented early in drug discovery (including SGLT2 inhibitor), lead optimization to reduce attrition at later stages of drug development.

Traditionally, radiolabeled substrates such as 2-deoxy-d-[^14^C]glucose, 2-deoxy-d-[^3^H]glucose or C-labeled α-methyl-d-glucopyranoside ([^14^C]AMG), are commonly applied to measure glucose uptake in screening of SGLT2 inhibitors [23, 24]. Although useful, but using the radioactive indicators has brought about many problems, such as the radioactive waste, radioactive cleanup, and restraint to licensed laboratories. 2-[*N*-(7-nitrobenz-2-oxa-1,3-diazol-4-yl) amino]-2-deoxy-d-glucose (2-NBDG) is a new fluorescent derivative of d-glucose which is modified at the C-2 position of the glucose with small fluorescent molecules 7-nitrobenz-2-oxa-1,3-diazol-4-yl chlorine [25]. This probe can be easily monitored for glucose through analysis of its fluorescence in living cells at a high-throughput single-cell level in vitro and in vivo [26, 27].

Given the promising future of SGLT2 inhibitors in treatment of T2DM, here we introduced a new approach to non-radioactively quantify glucose mediated by SGLT2 in HK-2 cells. The nonradioactive microplate and microscope-based high-throughput screening approach for glucose measurement is a new and alternative method for screening of SGLT2 inhibitor and implied for other cell assays of glucose measurement extensively.

## Results and Discussions

### Endogenous SGLT2 of HK-2 Cells Mediates Glucose Uptake

To study whether HK-2 cells are capable to uptake the SGLT2-mediated glucose influx, HK-2 cells, identified by selectively and highly expressed SGLT2 as previous reported (supplemental Fig. 1) [20], and the fluorescent glucose (2-NBDG) were applied for glucose measurement in our assay.

**Fig. 1:**
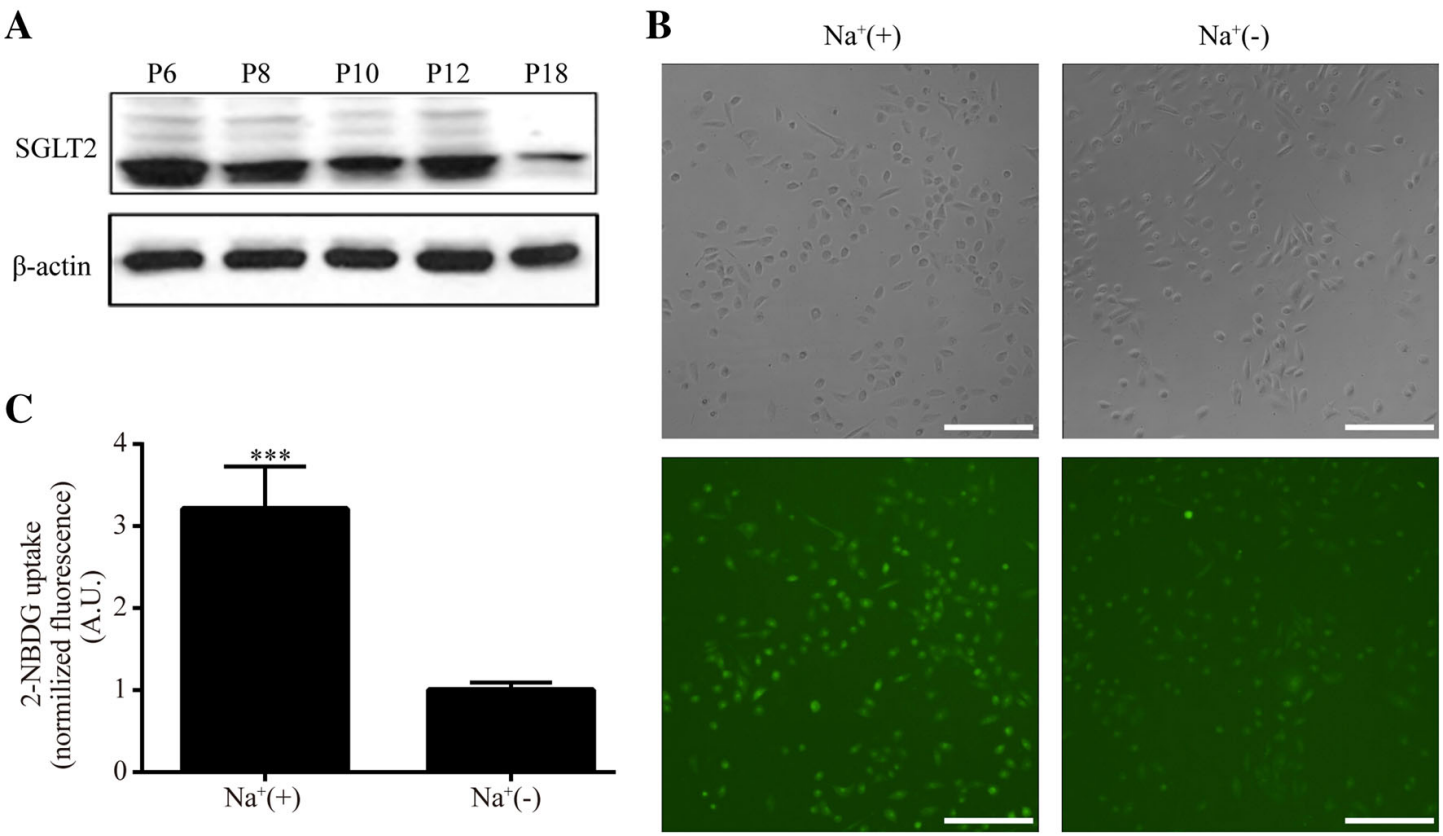
Endogenous SGLT2 of HK-2 cells mediates glucose uptake. **a** Western blot of SGLT2 expressions in serial passages of HK-2 cells. **b** HK-2 cells were incubated in Na^+^ (left) or Na^+^-free (right) buffer for 30 min with 200 μM 2-NBDG. Both the bright field images and the fluorescent images were taken at 10× magnification of objective. Na^+^(+), sodium buffer; Na^+^(−), sodium free buffer. Scale bar, 100 μm. **c** Quantification of 2-NBDG fluorescence intensity. The 2-NBDG values were normalized to the fluorescence intensity of Na^+^(−) group. Experiments were performed in triplicate and data are presented as mean ± S.E.M. of three independent experiments. A.U., arbitrary units. The significance was determined by two-tailed paired *t* test (***P < 0.001 vs. Na^+^(−) group)

For capturing the best readout for measuring SGLT2-mediated glucose uptake in HK-2 cells, we screen SGLT2 expressions during cell culturing up to passage 18. As shown in Fig. 1a, the SGLT2 expressions were constantly stable from passage 6 to passage 12, but its expression was significantly decreased at passage 18 (Fig. 1a), so the HK-2 cells used in current experiments were all finished before the passage 12.

HK-2 cells express two classes of glucose carriers, SGLT2 and facilitative glucose transporters 2 (GLUT2). SGLT2-mediated glucose transport is electrogenic, and coupled with Na^+^ transport at a stoichiometric ratio of 1:1 [28]. So Na^+^ is necessary for the glucose transport, whereas GLUT2-mediated glucose transport is via facilitative diffusion [29]. In current study, sodium buffer and sodium free buffer was used for cell incubations to measure 2-NBDG uptake. 2-NBDG transported in presence of Na^+^ is considered as the total uptake, which is the sum of contributions from SGLT2 and GLUT2, whereas the glucose uptake through GLUT2 alone were determined by incubating the cells in Na^+^-free buffer [29]. Based on previous experiments using 2-NBDG [29], the HK-2 cells were exposed to 200 μM 2-NBDG in Na^+^ buffer (Na^+^(+)) or Na^+^-free buffer (Na^+^(−)) respectively for 30 min and then washed and imaged by fluorescence microscope. As shown in Fig. 1b, the cells incubated with Na^+^(+) displayed a stronger fluorescence of 2-NBDG than the cells incubated in Na^+^(−). Then we analyzed and calculated the average fluorescence intensity of single cell in every group with MetaMorph software. The enhanced portion (~3.2 fold) of fluorescence by adding Na^+^ in the buffer compared to that of in Na^+^-free was implied as the Na^+^-dependent glucose uptake specifically mediated by SGLT2 as quantified in Fig. 1c.

Several cell lines currently used for SGLT2 inhibitor screening are pig kidney epithelial cells (LLC-PK1), primary monkey kidney cells (PMKCs), or COS-7/CHO cells that overexpress hSGLT2 [29–32]. However, these cells have substantial difference with human cells. It has been reported that human SGLTs shows differences in the kinetics and substrate specificities with other species, such as rabbit and rat [33]. And COS-7/CHO cells don’t have the characteristics of epithelial cells. The compounds screened out via these non-human and non-renal epithelial cell models mentioned above have lower success rate for developing anti-diabetic drugs. HK-2 cells could express SGLT2 normally and have the most original characteristic of proximal tubular cell, which thus would be more appropriate to develop into a model applied for SGLT2 inhibitor screening than the aforementioned cell lines.

### 2-NBDG Uptake in HK-2 Cells is Transported via SGLT2

Next, to evaluate 2-NBDG uptake in above assay specifically transported by glucose transporters, the competition experiments were performed by mixed treatment of cells with d-glucose and 2-NBDG.

**Fig. 2:**
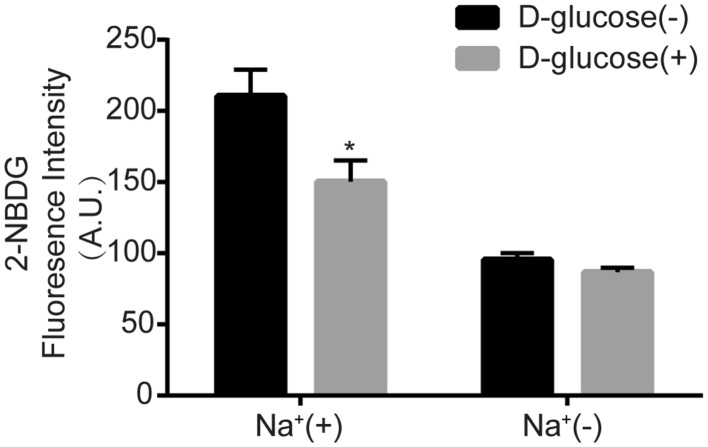
2-NBDG uptake is transported via SGLT2. Quantification of 2-NBDG fluorescence of HK-2 cells treated with or without sodium or d-glucose. Results of three independent experiments are presented as mean ± S.E.M. The significance was determined by two-tailed paired *t*-test (*P < 0.05 vs. Na^+^(+) group without d-glucose)

The Na^+^-dependent glucose uptake was measured in cells incubated with 2-NBDG (200 μM) alone or with d-glucose (30 mM). As showing, the 2-NBDG uptake was about 210.6 ± 36.9 A.U. in the absence of d-glucose, whereas its level decreased to 150.4 ± 29.8 A.U. when d-glucose was present, supporting the presence of glucose as a competitor reduced 2-NBDG uptake (Fig. 2, Na^+^ (+) groups). On the other hand, d-glucose supplement had no significantly effect on 2-NBDG uptake in Na^+^-free buffer (Fig. 2, Na^+^(−) groups). The results support that competition by glucose was specific to the Na^+^-dependent uptake of 2-NBDG, and 2-NBDG is fitted for the glucose constitute in measurement of glucose.

### Optimization of Incubation Time and Concentration of 2-NBDG

**Fig. 3:**
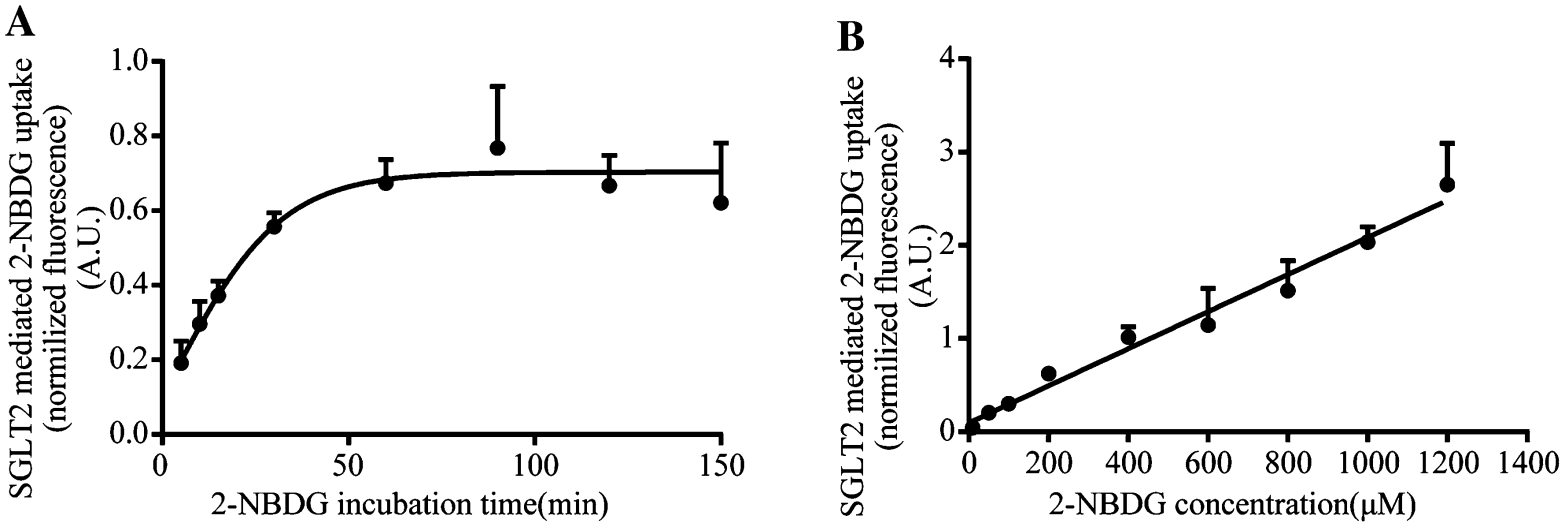
Optimization of incubation time and concentration of 2-NBDG. **a** HK-2 cells were incubated with 200 μM 2-NBDG for 5, 10, 15, 30, 60, 90, 120 or 150 min in Na^+^(+) or Na^+^(−) buffer. SGLT2-mediated 2-NBDG uptake was calculated by subtracting the intensity of the Na^+^(+) to Na^+^(−) group. **b** HK-2 cells were incubated with 10, 50, 100, 200, 400, 600, 800, 1000 or 1200 μM 2-NBDG for 60 min in Na^+^ (+) or Na^+^ (−) buffer, and then SGLT2-mediated 2-NBDG uptake was calculated as above. Experiments were performed in triplicate and data are represented as mean ± S.E.M. of three independent experiments

For the best fluorescence of 2-NBDG detected by microscope, HK-2 cells were incubated with 200 μM 2-NBDG in Na^+^ or Na^+^-free buffer for 5, 10, 15, 30, 60, 90, 120 or 150 min respectively, followed by measuring the fluorescence intensity of 2-NBDG in the cells. The quantification in Fig. 3a reflected the increasing glucose uptake in HK-2 cells in the processing of the more incubation time. Particularly, the glucose uptake reached the maximum by the 60 min of the 2-NBDG incubation.

Under this condition, 2-NBDG was serially diluted to 10, 50, 100, 200, 400, 600, 800, 1000 and 1200 μM in the presence or absence of Na^+^ with the 60 min incubation time and then measured the glucose uptake. The data demonstrated that glucose uptake in HK-2 cells is linearly correlated the doses up to 1200 μM of 2-NBDG (Fig. 3b). Given that higher concentrations of 2-NBDG led to larger variations, which may be caused by the fluorescence quenching of 2-NBDG in the process of taking photos and self-quenching of 2-NBDG at higher concentrations [34]. Therefore, 200 μM is well applied for our glucose uptake assay.

Most studies on glucose uptake are commonly carried out using radiotracers such as 2-deoxy-D-[^14^C]glucose, 2-deoxy-d-[^3^H]glucose or [^14^C]AMG [26]. However, there are several disadvantages associated with the radiotracer such as disposal of radioactive waste or radioactive cleanup [31]. More importantly, it cannot directly measure glucose uptake in single, living cells. Regarding these aspects, 2-NBDG, fluorescence at 542 nm when excited at 467 nm [35, 36], is a well-designed probe which can direct measurement of glucose uptake [35]. Currently we showed the application of 2-NBDG is a practical and simple cell-based method and can reflect single cell fluorescence (Figs. 1–3) based on the analysis of average fluorescence intensity of single cell in every group.

### Effects of Starving HK-2 Cells on Glucose Uptake

**Fig. 4:**
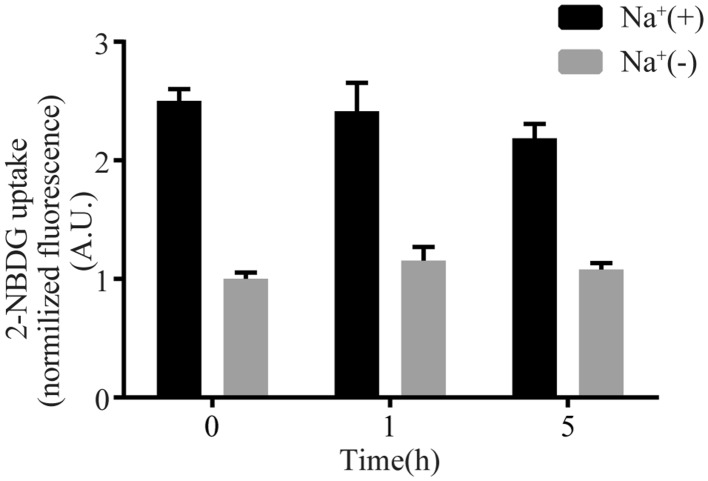
Effects of Starving HK-2 cells on glucose uptake. HK-2 cells were incubated in DMEM without glucose for 0, 1 and 5 h, then 2-NBDG uptake assay was performed in Na^+^(+) or Na^+^(−) buffer. The uptake values were normalized to the fluorescence intensity of Na^+^(−) group without starvation. Experiments were performed in triplicate and data are expressed as mean ± S.E.M of three independent experiments

Prior to the glucose uptake measurement, moderate starvation without glucose supplements results in an increased glucose uptake into cells, in general. To optimize the best readout for glucose uptake of the HK-2 cells, we next incubated the cells in glucose-free DMEM for 0, 1 h or 5 h before adding the buffer supplemented with 2-NBDG, and then followed by the treatment with regular buffer for our glucose uptake assay as in Fig. 4. Surprisingly, both total glucose uptake (Na^+^(+) and Na^+^-independent (Na^+^(−)) glucose uptake are relatively stable among 0, 1 h and 5 h starvations of the cells, suggesting no starvation is required for the SGLT2-mediated glucose uptake assay in HK-2 cells.

### SGLT2 Inhibitors Dapagliflozin and Phlorizin Block 2-NBDG Uptake in HK-2 Cells

**Fig. 5:**
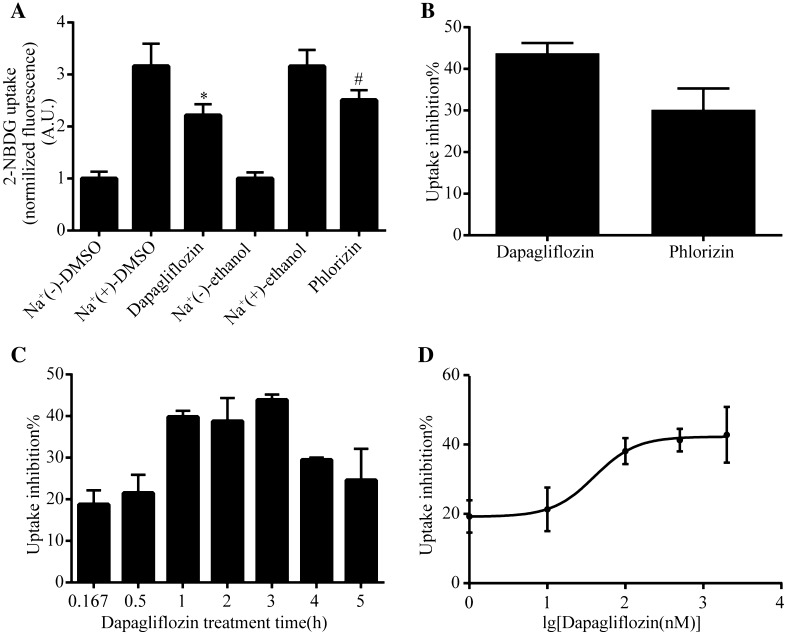
SGLT2 inhibitors Dapagliflozin and Phlorizin blocks 2-NBDG uptake in HK-2 cells. **a** Inhibitory effect of Dapagliflozin (500 nM) or Phlorizin (100 μM) on 2-NBDG uptake in HK-2 cells. Values were normalized to the fluorescence intensity of 2-NBDG uptake dissolved in Na^+^(−) buffer. Experiments were performed in triplicate and data are presented as mean ± S.E.M. of three independent experiments. *P < 0.05 versus Na^+^(+)-DMSO; #P < 0.05 versus Na^+^(+)-ethanol. **b** Inhibition ratio of Dapagliflozin and Phlorizin derived from (**a**). **c** 2-NBDG uptake was measured in HK-2 cells incubated with Dapagliflozin for 0.167, 0.5, 1, 2, 3, 4 and 5 h. **d** Dose-dependent response curve of Dapagliflozin on 2-NBDG uptake assay of HK-2 cells

To address the inhibition effects of SGLT2 inhibitors on 2-NBDG uptake in our screening assay is reliable and similar to traditional methods, we treated HK-2 cells with two SGLT2 inhibitors Dapagliflozin and Phlorizin and then performed the 2-NBDG uptake assay. As we predicted, Dapagliflozin (500 nM) strongly inhibited Na^+^-dependent glucose uptake by reducing 43.7% of 2-NBDG uptake, whereas Phlorizin (100 μM) induced 30.2% decrease in Na^+^-dependent glucose uptake (Fig. 5a, b). These positive results constitute a good starting point for assay development.

Furthermore, we measured the inhibition of 2-NBDG uptake by Dapagliflozin in a serial time points (0.167, 0.5, 1, 2, 3, 4 and 5 h, Fig. 5c) and incremental concentrations (1, 10, 100, 500 and 2000 nM, Fig. 5d) conditions. We observed that the percentage inhibition of Dapagliflorin on 2-NBDG uptake were increased as treatment time increased, and the 1, 2 and 3 h incubation with the drug reached the maximal inhibition (Fig. 5c). Similarly, the percentage of inhibition by the drug on glucose uptake was dose-dependent in the range from 1 to 2000 nM (Fig. 5d), which is consistent as previous reports of the drug [31].

It is well known that non-radioactive assays are in general less sensitive than radioactive ones, however, the safer processes and cheaper substrate used, in our opinion, greatly preponderate this disadvantage. Compared to the well-known [^14^C]-AMG radioactive assay, 2-NBDG based screening assay is low cost, easy-to-handle, more feasible and more stable. Our new assay system with safer processes and a cheaper substrate can be used to primary screening for SGLT2 inhibitors, radioactive assay can then be applied to the secondary screening assay to confirm the inhibitory activity and further optimization of candidate compounds.

Present SGLT2 inhibitors belong to a class of structurally-related aromatic C- and O-β-d-glucopyranoside analogues of the natural product phlorizin. Phlorizin, T-1095A, sergliflozin and remogliflozin are *O*-glycosides. Dapagliflozin is C-glycoside SGLT2 inhibitor. The two sets of compounds exhibit excellent glucose uptake inhibitory activity in this assay (Fig. 5). Only few reports have reported the discovery of nonglycoside inhibitors, such as flavonoid antioxidants and cyclic diarylheptanoids [37, 38]. Identification of non-glycoside inhibitors could be advantageous, which will lay the foundation for chemical structure of new-type SGLT2 inhibitor and make it pluralistic. Further application of this system in high throughput screening will help to find new SGLT2 inhibitors for diabetes treatment.

Together, present assay for fluorescently measuring SGLT2-mediated glucose uptake in HK-2 cells is a sensitive, effective, easy-operated, nonradioactive and physiological approach as an alternative method for screening SGLT2 inhibitors.

## Experimental Section

### Reagents

Keratinocyte-SFM (serum-free medium) was obtained from Gibco (Grand Island, NY, USA). 2-NBDG was from Invitrogen (Carlsbad, CA, USA). Dapagliflozin and Phlorizin were purchased from Selleck (USA), *N*-Methyl-d-glucamine and all other chemicals were from Sigma-Aldrich (St. Louis, MO, USA).

### Cell Culture

Cell culture plates were from Corning-Costar (Corning, NY, USA). HK-2 cells were purchased from the American Type Culture Collection (Manassas, VA, USA) and were grown in Keratinocyte-SFM supplemented with human recombinant Epidermal Growth Factor (rEGF), Bovine Pituitary Extract (BPE), 0.025 g/L penicillin and 25 mg/mL streptomycin. Cells were plated in 60-mm diameter culture plates and were maintained at 37 °C in a 5% CO_2_ atmosphere incubator.

### Western Blot

HK-2 cells grown in a 35-mm plates were rinsed with ice-cold phosphate buffered saline, PBS (137 mM NaCl, 8 mM NaH_2_PO_4_, 2.7 mM KCl, 1.5 mM KH_2_PO_4_; pH 7.4). While kept on ice, 0.2 ml of RIPA Lysis Buffer (Beyotime, China) was added to the plates and the cells were scraped off. The lysate was then vortexed and incubated on ice for 30 min. Lysed cells were loaded and resolved on a 5% stacking and 10% running SDS-PAGE, and the proteins were transferred to PVDF membrane. Antibody incubations and non-specific binding sites were blocked with 5% skim milk. The membranes were incubated with primary antibodies SGLT2 (Abnova, Taipei, Taiwan) and β-actin (Cell Signaling Technology, Danvers, MA) diluted in 5% skim milk in TBS-T, and incubated with horseradish peroxidase-conjugated anti-mouse secondary antibody. The bands were visualized with chemiluminescence (Pierce ECL, Thermo, Waltham, MA).

### 2-NBDG Uptake Measurement

Cells were plated at 1 × 10^4^/well of the 96-well plates and used at sub-confluence after 24 h pre-incubation. Sodium buffer (Na^+^ buffer), prepared for cell incubations in the presence of sodium conditions, contained 140 mM NaCl, 5 mM KCl, 2.5 mM CaCl_2_, 1 mM MgSO_4_, 1 mM KH_2_PO_4_, and 10 mM HEPES (pH 7.4). Sodium free buffer (Na^+^-free buffer) contained 140 mM *N*-Methyl-d-glucamine instead of NaCl and was used for cell incubations to measure 2-NBDG uptake in the absence of sodium.

For experiments, all culture medium was removed from each well and rinsed in Na^+^-free buffer for three times. HK-2 cells were then incubated in 100 μl Na^+^ or Na^+^-free buffer in the absence or presence of 2-NBDG together with compounds at indicated concentrations. Plates were incubated at 37 °C with 5% CO_2_ for a period of time as described in each experiment. The 2-NBDG uptake reaction was stopped by removing the incubation medium and washing the cells three times with pre-cold Na^+^-free buffer. Auto-screening inverted epifluorescence microscopy (Nikon Ti-E) fitted with an EM CCD (ANDOR iXon3) for imaging of fluorescent 2-NBDG with the filter for 488-nm excitation and 545-nm emission. The 20 × objective lens was used to provide both high light transmission and a wide optical field to allow for simultaneous imaging of multiple cells in the same field. This required that the fluorophore must first be flushed from the extracellular media before imaging. For each experiment, the fluorescence output from 200 to 600 cells was collected, digitized and stored on the computer workstation for analysis.

2-NBDG in cells in the presence of Na^+^ is considered as the total glucose uptake, which is the sum of contributions from SGLTs and GLUTs. To determine transport mediated only by GLUTs, cells were incubated for a period of time with 2-NBDG in Na^+^-free buffer. The difference between the total and the Na^+^-independent uptake was calculated and defined as Na^+^-dependent glucose uptake mediated by SGLT2 [29].

### Statistical Analysis

Unless otherwise stated, at least three independent repeat were conducted for each experiment. The images captured with auto-screening inverted epifluorescence microscopy contained 200–600 cells. Imaging analysis was performed by MetaMorph software (Molecular Devices, Sunnyvale, CA). Briefly, the thresholding value was determined manually at first, then single cell was designated by defining morphometric parameters, shape and size. Created the regions and objects and the values of average fluorescence intensity quantified from single cell were obtained. After background subtraction, fluorescence intensity was calculated as the difference in the average fluorescence of cells in absence or presence of Na^+^. The mean values of the results were calculated, and standard error of mean (S.E.M.) values were determined. For statistical analysis, comparisons between multiple groups were performed using two-tailed paired *t* test.

## Conclusions

In summary, we developed a non-radioactive and physiological method to measure glucose transport-mediated by SGLT2 in cultured HK-2 cells using fluorescent glucose (2-NBDG), which could be used for high-throughput screening of SGLT2 inhibitors. The method presented here is more convenient, cost-saving and pollution-free than traditional assays.

## Electronic supplementary material

Below is the link to the electronic supplementary material.

Supplementary material 1 (PDF 283 kb)
