# Supplementary material for: A Fluorescent Glucose Transport Assay for Screening SGLT2 Inhibitors in Endogenous SGLT2-Expressing HK-2 Cells
Source: Nat Prod Bioprospect. 2018 Nov 1;9(1):13–21. doi: 10.1007/s13659-018-0188-4 (PMC6328422; doi:10.1007/s13659-018-0188-4)
Supplement: Supplementary file 1 — Supplementary material 1 (PDF 283 kb) [file 13659_2018_188_MOESM1_ESM.pdf]

**Supplemental Material**

# A Fluorescent Glucose Transport Assay for Screening SGLT2 Inhibitors in endogenous SGLT2-expressing HK-2 cells


Yanting Lu[1,2], Xiuli Ma[1,2], Yuhui Xu[1,2], Jing Hu[1,2], Fang Wang[1,2], Wanying Qin[1,2], Wenyong Xiong[1,3]

[1] State Key Laboratory of Phytochemistry and Plant Resources in West China, Kunming Institute of Botany, Chinese Academy of Sciences, Kunming 650201, China

[2] University of the Chinese Academy of Sciences, Beijing 100049, China

[3] Yunnan Key Laboratory of Natural Medicinal Chemistry, Kunming 650201, China

Correspondence: Wenyong Xiong, State Key Laboratory of Phytochemistry and Plant Resources in West China, Kunming Institute of Botany, Chinese Academy of Sciences, Kunming, China. Tel: 86-871-65216750. Email: xiong.wenyong@mail.kib.ac.cn


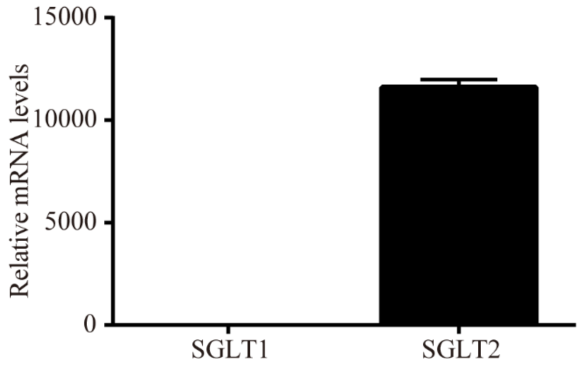


**Supplemental Fig. 1** SGLT2 highly expressed in HK-2 cells. Q-PCR analysis of gene expression in HK-2 cells.
